# Structural, electronic and magnetic properties of M_x_Pt_1-X_, (M= Co, Ni and V) binary alloys

**DOI:** 10.1016/j.heliyon.2019.e02433

**Published:** 2019-09-10

**Authors:** A.M. Alsaad, A.A. Ahmad, Hamzah A. Qattous

**Affiliations:** a Department of Physical Sciences, Jordan University of Science & Technology, P.O. Box 3030, Irbid, 22110, Jordan; b Department of Physics, Faculty of Sciences, University of Hafr Al-Batin, Saudi Arabia

**Keywords:** Materials science, Physics, Condensed matter physics, Density functional theory (DFT), Vienna ab initio simulation package (VASP), Density of states (DOS), Magneto-crystalline anisotropy energy (MAE), Electronic properties, Computational materials science, Materials application, Materials physics, Materials property, Materials structure

## Abstract

The structural, optical and magnetic properties of ordered M_x_Pt_1-x_ (M = Co, Ni and V) binary alloys have been investigated using Vienna ab initio Simulation Package (VASP) within the framework of Density Functional Theory (DFT) and the Generalized Gradient Approximation (GGA). *Ab initio* calculations have been performed to obtain the most stable structure for each of the three binary systems. In addition, the optical and electrical properties such as electronic band structure, density of states and partial density of states of M_x_Pt_1-x_ binary alloys have been investigated. Specifically, total energy minimization has been performed to calculate the equilibrium in-plane, *a*_*o*_, out-of-plane, *c*_*o*_, and volume, *V*_*o*_, structural lattice parameters of M_x_Pt_1-x_ binary alloys. We found that *a*_*o*_, *c*_*o*_ and *V*_*o*_ for CoPt, NiPt and VPt_3_ equal to (*a*_*o*_ = 3.806 A, *c*_*o*_ = 3.707 A and *V*_*o*_ = 53.7 A^3^) (*a*_*o*_ = 3.84 A, *c*_*o*_ = 3.62 A and *V*_*o*_ = 53.64 A^3^) and (*a*_*o*_ = 3.88 A, *c*_*o*_ = 7.88 A and *V*_*o*_ = 118.71 A^3^) respectively. Furthermore, the magneto-crystalline anisotropy energies (MAE) have been calculated to get a deeper insight into magnetic characteristics of the M_x_Pt_1- x_ binary alloys. We found that MAE values for CoPt, NiPt and VPt_3_ binaries are equal to 1.60, 0.231 and 0.0116 meV/unit cell respectively. These MAE values correspond to magneto-crystalline anisotropy constant K values equal to 4.8 ×10^7^, 6.9 ×10^6^ and 1.46 × 10^5^ erg/cm^3^. The obtained results reveal that CoPt and NiPt binary systems exhibit attractive optical and magnetic properties, which make both systems potential candidates for magneto-optical and optical-electronic devices. Our results are in good agreement with the previous experimental and theoretical findings.

## 1 Introduction

Magnetic materials based on transition metals such as cobalt (Co) and nickel (Ni) and their chemical binary compounds are extremely important because of their potential applications in optical filters [1], data storage [2], magnetic recording media [3] and biomedical applications [4, 5]. Recently, materials obtained from alloying transition metals and Platinum (Pt) are very important class of magnetic materials, due to their potential to be good medium materials for high-density magnetic recording [6]. The platinum-group metals (PGMs) binary alloys have attracted a lot of interest because of their main role in a wide variety of industrial applications [7], aeronautics [8] and electronics applications [9]. The importance and high cost of PGMs causes many efforts motivated to usage that is more effective or at the development of less-expensive alloy substitutes. The capability to identify new phases is the key in tuning the structural, electrical and, magnetic properties of PGM alloys and their utilization in new applications, as reduced-cost or higher-activity substitutes in current applications. Compared to other binary alloys, the transition metals-platinum binary alloys such as (FePt, CoPt and MnPt) show excellent mechanical, optical and magnetic properties. Furthermore, they are recognized to exhibit high coercivity and large magneto-crystalline anisotropy energies (MAE) [10, 11]. Combination between the Pt element and the 3d transition metal indicate promising magnetic properties [5, 12]. Specifically, multi-layers of Co and Pt now intensively studied due to their potential usage in magneto-optical storage devices as optical storage material [13, 14, 15]. The accurate calculation of MAE is very critical in designing and fabricating of devices for density data storage, high-density magnetic recording applications [16, 17]. Furthermore, MAE value gives an indication about the magnetic stability of the structure in response to the orientation of the magnetic spin moment. Several approaches have been proposed for the accurate calculation of MAE parameter in magnetic based-compounds because this parameter determines how these compounds would be integrated into magneto-optical devices.

**Fig. 1:**
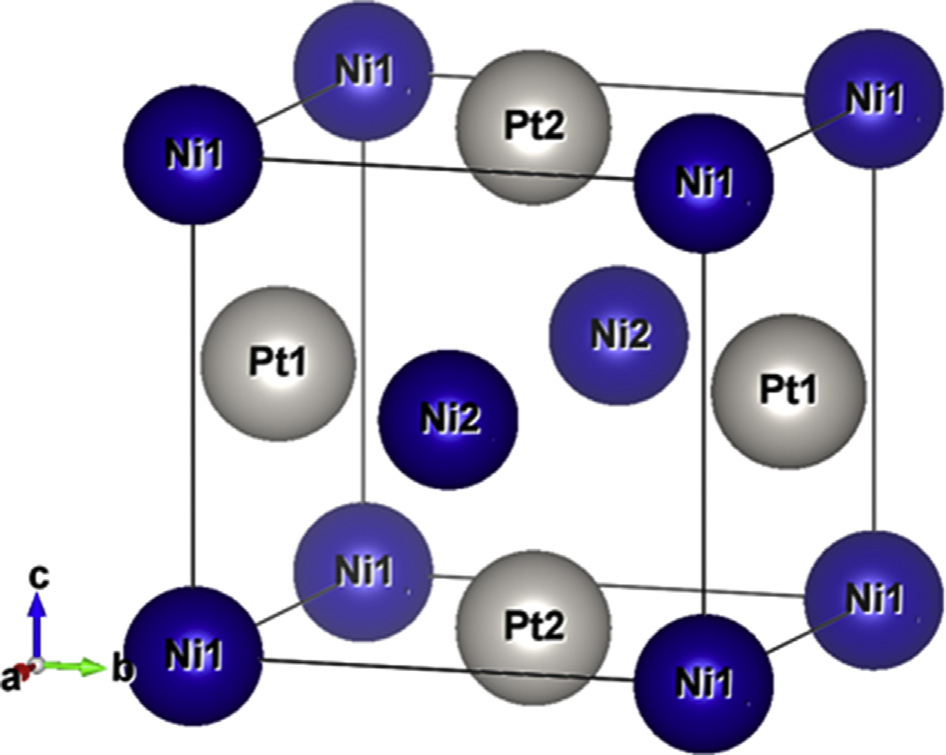
L1_0_ structure of (a) CoPt and (b) NiPt unit cell.

Ordered binary alloys of CoPt and NiPt at *50:50* composition exhibit face-centered tetragonal (fct) (L1_0_) phase as illustrated in Fig. 1.

The L1_0_ structure recognized as a derivative of the face centered cubic (fcc) structure. In L1_0_ structure one type of atoms occupy two of the faces and corners and the second type of atoms occupy the other faces. The ratio *c/a* is an important property of this structure, in this phase; both systems are expected to have excellent magnetic properties.

**Fig. 2:**
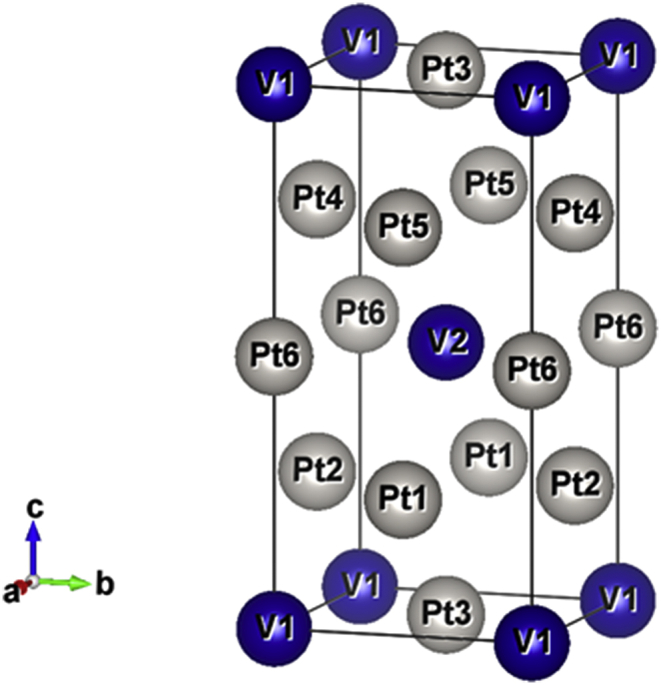
The tetragonal (TiAl_3_- like) crystalline structure of VPt_3_ unit cell.

The VPt_3_ structure generally exists in ordered states with fcc Cu_3_Au-like and tetragonal TiAl_3_- like crystalline structures, In the present work, the tetragonal structure will be investigated. Fig. 2 shows the VPt_3_ tetragonal structure.

In the present work, we computed the optimized lattice parameters of M_x_Pt_1-x_ (M = Co, Ni and V) using DFT-based total energy minimization approach. The electronic band structure, density of states and partial density of states of M_x_Pt_1-x_, (M = Co, Ni and V) have been computed and reported. The force theorem has been implemented to accurately compute the MAE of M_x_Pt_1-x_, (M = Co, Ni and V) binaries.

## 2 Methodology

In this section, we present a brief introduction to the Vienna ab initio Simulation Package (VASP) that is installed on the clusters of the Holland Computing Center (HCC) affiliated by the University of Nebraska. In addition, we describe the research methodology and introduce the computational details that have been used to obtain the results of this work, such as total energy minimization, magneto-crystalline anisotropy energy, density of states and band structure data. The calculations were performed within the framework of Density Functional Theory (DFT) and implementing the Local Density Approximation (LDA) [18], the Generalized Gradient Approximation (GGA) [19, 20, 21] and the Projected-Augmented Plane- Wave PAW [22, 23], as implemented in the VASP code. Vienna Ab initio Simulation Package (VASP) is a software package used for electronic structure calculations and quantum-mechanical molecular dynamics, from first principles [24]. VASP computes an approximate solution to the many-body Schrödinger equation, within DFT. In addition, VASP uses post- DFT corrections. For example, Hybrid functional that mix the Hartree-Fock approach with DFT, many-body perturbation theory and dynamical electronic correlations within the random phase approximation are implemented as well [25].

## 3 Results and discussion

### 3.1 Total energy minimization and optimized atomic configurations

The structural optimization of given structure is important for further investigations of it is properties. The self-consistency cycle (SC) seems to be the most efficient way for calculating the KS-ground state of metallic systems [26, 27]. Total energy versus the volume of unit cell of M_x_Pt_1-x_, (M = Co, Ni and V) binaries has been computed in order to obtain the optimized lattice parameters [28, 29].

For CoPt and NiPt, we performed self-consistent calculations with uniform k point grid according to the Monkhorst-Pack scheme with size of 8 × 8 × 8, whereas for VPt_3_ the k-points mesh was generated with a grid size of 9 × 9 × 6. The cut-off energy was taken to be 400 eV and the PAW pseudopotentials [30, 31] include the following valence electrons: Co: 3d^7^ 4s^2^, Ni:3d^8^ 4s^2^, V: 3d^3^ 4s^2^ and Pt: 5d^9^ 6s^1^. The optimized structures of all compounds investigated in this work were obtained by minimizing the total energy until the changes in energy between two consecutive SCF steps were less than 0.001 meV and the Hellmann–Feynman forces acting on each atom was reduced to be less than 0.0002 eV/Å.

The total energy minimization approach has been used to obtain the optimized structural parameters (equilibrium volume (*V*_*o*_), in-plane lattice constant (*a*_*o*_), out-off plane lattice constant (*c*_*o*_) and the axial ratio (*c*_o_/*a*_o_)).

**Fig. 3:**
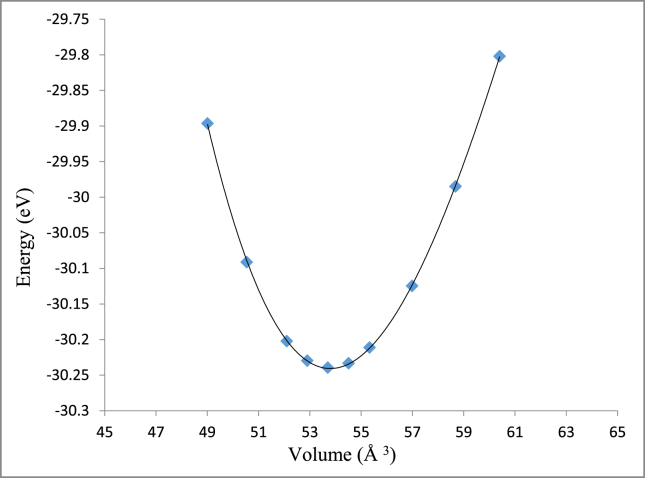
Total energy versus volume of a unit cell of CoPt binary.

**Fig. 4:**
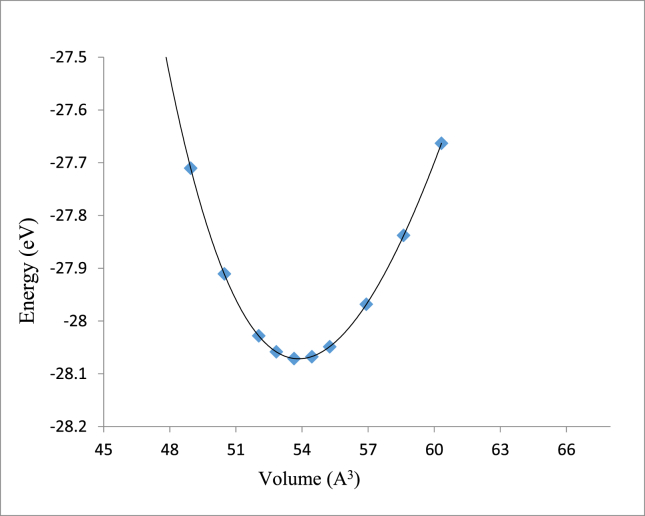
Total energy versus volume of a unit cell of NiPt binary.

**Fig. 5:**
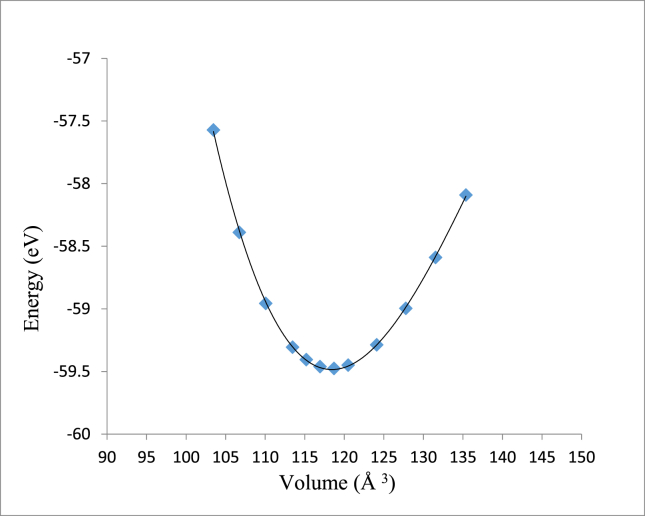
Total energy versus volume of a unit cell of VPt_3_ binary.

Both CoPt and NiPt exhibit the distorted face centered tetragonal phase with (P4/mmm) space group known as L1_0_ structure. The equilibrium volume can be written as: *V*_*o*_ = *a*_o_^2^ × *c*_*o*_, where *a*_*o*_ and *c*_*o*_ are the equilibrium lattice constants. By plotting the total energy versus the unit cell volume as shown in Figs. 3 and 4, the optimized structural parameters were extracted to be (*a*_*o*_ = 3.805, *c*_*o*_ = 3.707) A, *V*_*o*_ = 53.7 A^3^ and *c*_*o*_/*a*_*o*_ = 0.974 for CoPt binary and (*a*_*o*_ = 3.84, *c*_*o*_ = 3.62) A, *V*_*o*_ = 53.64 A^3^ and *c*_*o*_*/a*_*o*_ *=*0.9427 for NiPt binary. The unit cell of VPt_3_ has the tetragonal structure with (I4/mmm) space group and its equilibrium volume can be written as: *V*_*o*_ = *a*_*o*_^2^ × *c*_*o*_, where ao and co are the equilibrium lattice constants. The optimized structural parameters obtained by plotting the total energy versus the unit cell volume as seen in Fig. 5. The optimized structural parameters obtained to be (*a*_*o*_ = 3.88, *c*_*o*_ = 7.88) A, *V*_o_ = 118.71 A^3^ and *c*_*o*_/*a*_*o*_ = 2.03. Our results on structural lattice parameters of the three investigated parameters are summarized in Table 1. Our results were found to agree well with the previous experimental and theoretical findings.

**Table 1:** Values of equilibrium lattice parameters of CoPt, NiPt and VPt_3_ binaries.

System	CoPt	NiPt	VPt_3_
a_o_ (Å)	3.806	3.840	3.880
a_o__th_ (Å)	3.803 [32]	3.814 [33]	3.870 [34]
a_o__exp_ (Å)	3.810 [35]	3.840 [35]	3.861 [36]
c_o_ (Å)	3.707	3.620	7.880
c_o__th_ (Å)	3.701 [32]	3.533 [33]	7.84 [34]
c_o__exp_ (Å)	3.710 [35]	3.610 [35]	7.842 [36]
c_o_/a_o_	0.974	0.942	2.030
c_o_/a_o__th_	0.971 [32]	0.926 [33]	2.027 [34].
c_o_/a_o__exp_	0.973 [35]	0.940 [35]	2.031 [36]

### 3.2 Density of states (DOS) and band structure calculations

Having obtained the optimized lattice parameters of all the investigated structures, electronic properties such as Density of States (DOS) and Band Structure were investigated to understand how electrons are arranged in each of the three investigated binaries. DOS can be defined as the number of states per unit energy per unit volume [37]. Band structure and density of states have been investigated using VASP within the framework of spin density functional theory and generalized gradient approximation (GGA). The following computational parameters have been used to obtain the DOS data: the cut-off energy was taken to be 400 eV, the k-points mesh was generated with a grid size of 18 × 18 × 18. For CoPt and NiPt binaries, the number of atoms per unit cell equal to four whereas, for VPt_3_ the number of atoms per unit cell equals to eight. The band structure can be calculated along the high symmetry points in Brillouin zone.

Analysis of partial DOS reveal that the contributions to total DOS of M_x_Pt_1-x_ (M = Co, Ni and V) is dominated mainly by M-3d and Pt-5d electronic states. Band structure indicates that all of M_x_Pt_1-x_ binary alloys are metallic in nature. In this section we present the calculated band structure, total and partial density of states of CoPt, NiPt and VPt_3_ binaries.

#### 3.2.1 Density of states and electronic band structure of CoPt systems

**Fig. 6:**
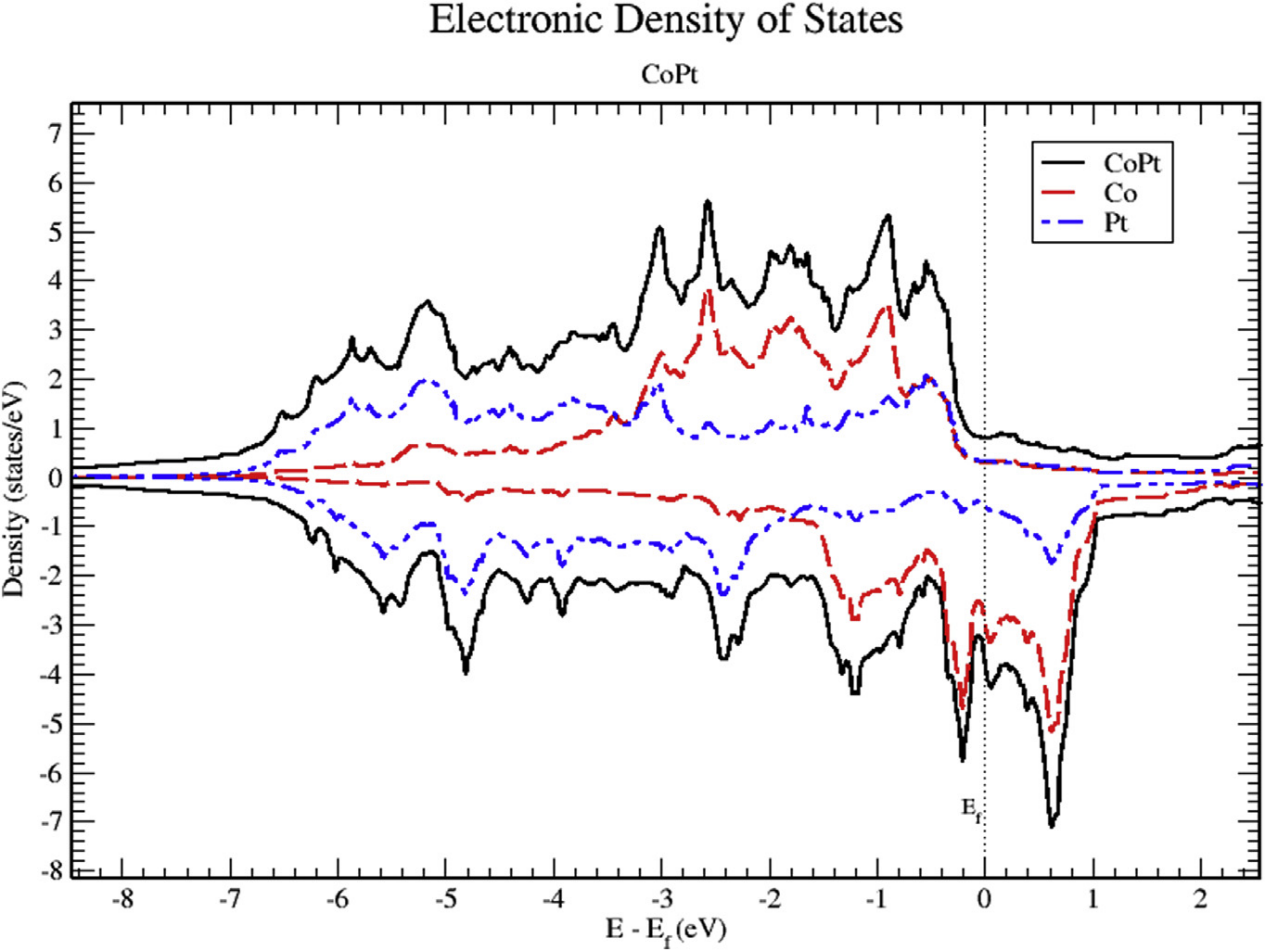
Total and partial density of states of CoPt binary, Co (red) and Pt (blue).

**Fig. 7:**
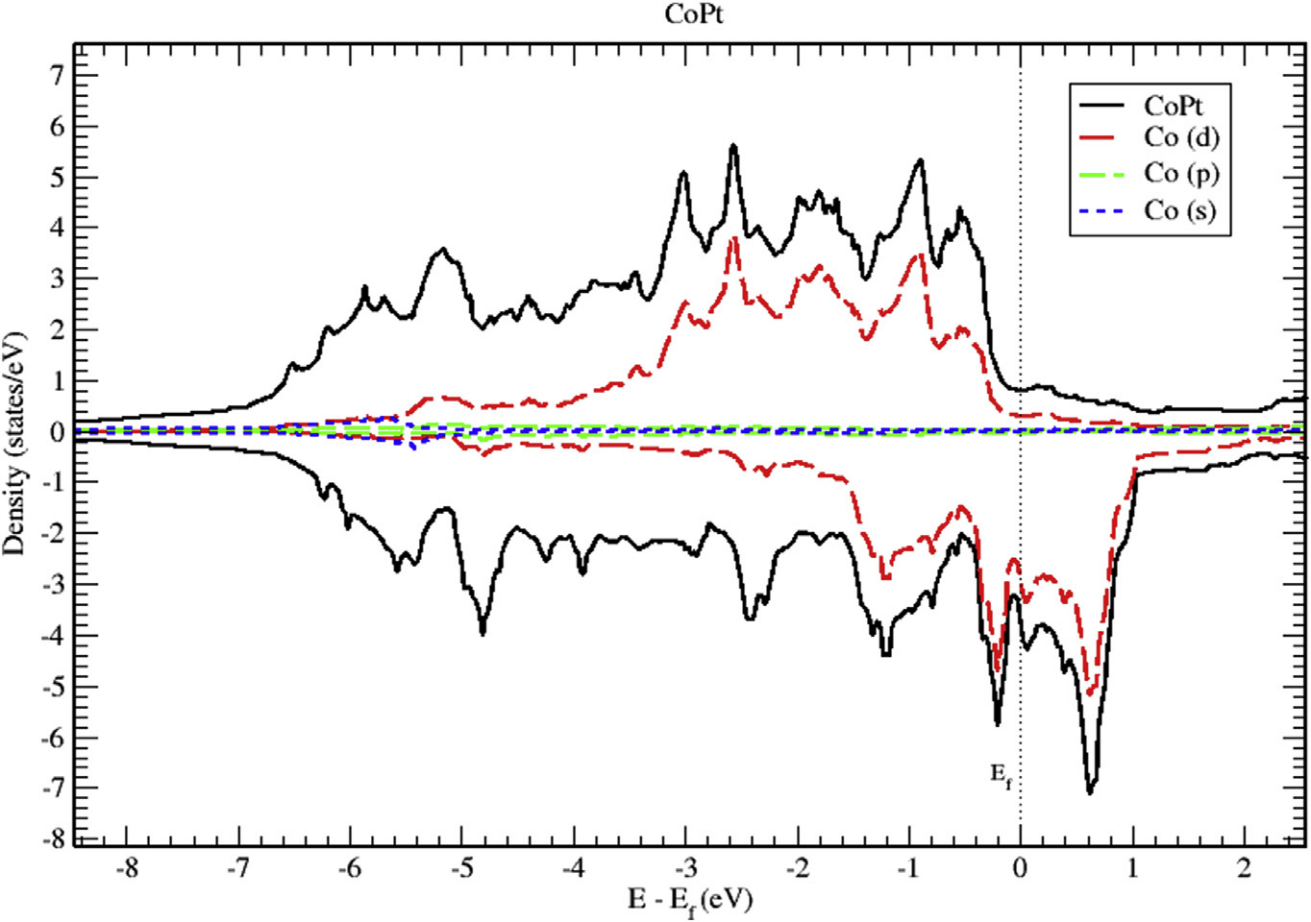
Total and partial density of states of CoPt binary, Co-d (red), Co-p (green) and Co-s (blue).

**Fig. 8:**
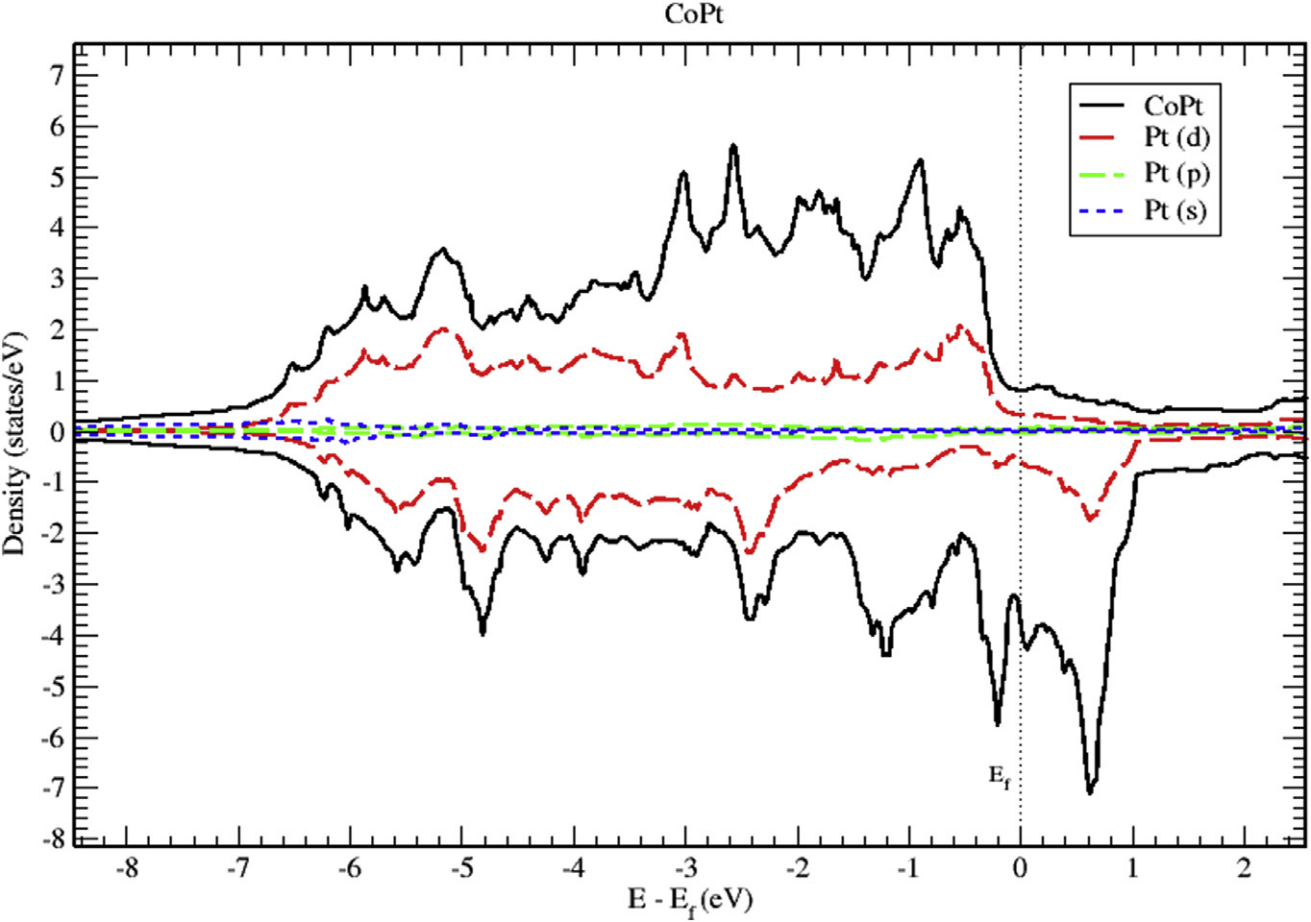
Total and partial density of states of CoPt binary, Pt-d (red), Pt-p (green) and Pt-s (blue).

Figs. 6, 7, and 8 show the total and the partial DOS plots of CoPt binary. The contributions to total DOS of CoPt have been found to come mainly from Co-3d and Pt-5d electronic states. The peak located at 0.6 eV above Fermi energy shows that the Co-3d electronic state dominates the conduction band, while the valence band is dominated by Co-3d and Pt-5d electronic states. The highest DOS of spin up valence bands are found to be located at (-0.55, -0.88, -2.56 and -3.02) eV below Fermi level, whereas, the highest DOS of the spin down valence bands found to be located at (-0.22, -1.19, -2.44 and -4.81) eV below Fermi level. The width of spin up band (5.95) eV is narrower than the spin down band (7.1) eV. The partial DOS of Co and Pt atoms show strong d-d hybridization below fermi level, such hybridization originates the ferromagnetic spin configuration and enhances the value of the magnetic moment. The large values of magnetic moment of CoPt binary make it eligible for a wide range of magnetic applications.

**Fig. 9:**
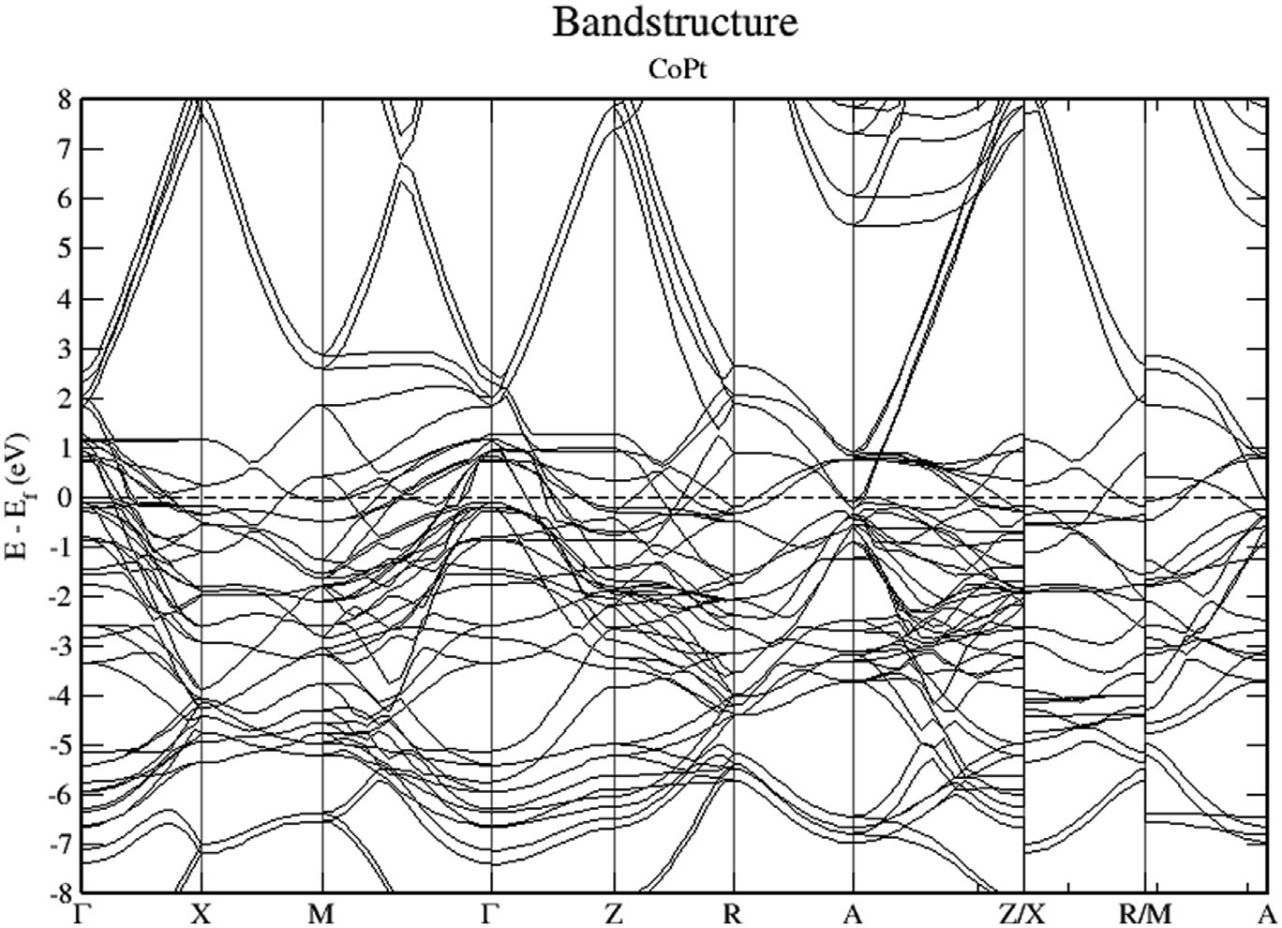
Electronic band structure of CoPt binary.

**Fig. 10:**
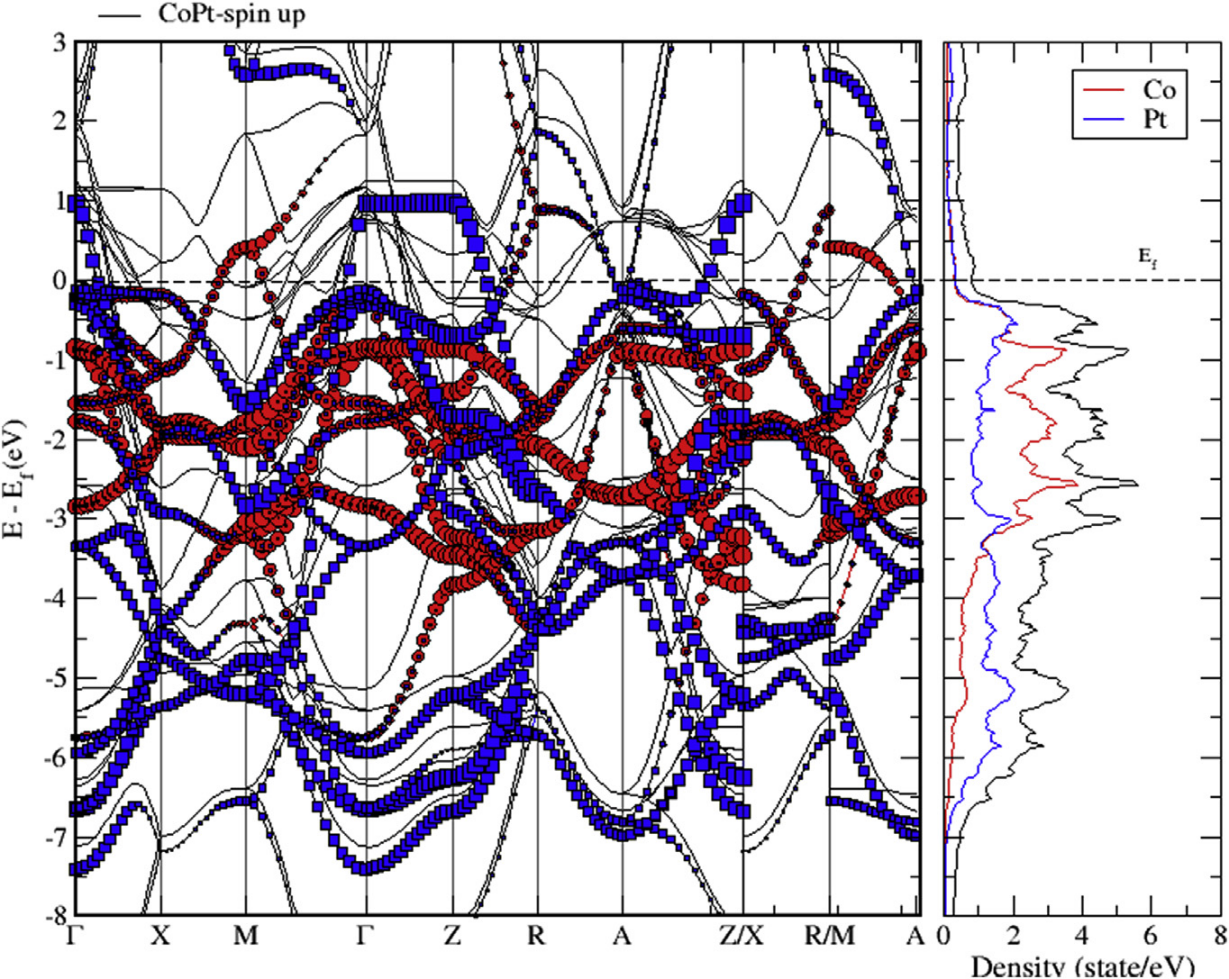
Electronic band structure and partial DOS of spin up states of CoPt binary.

**Fig. 11:**
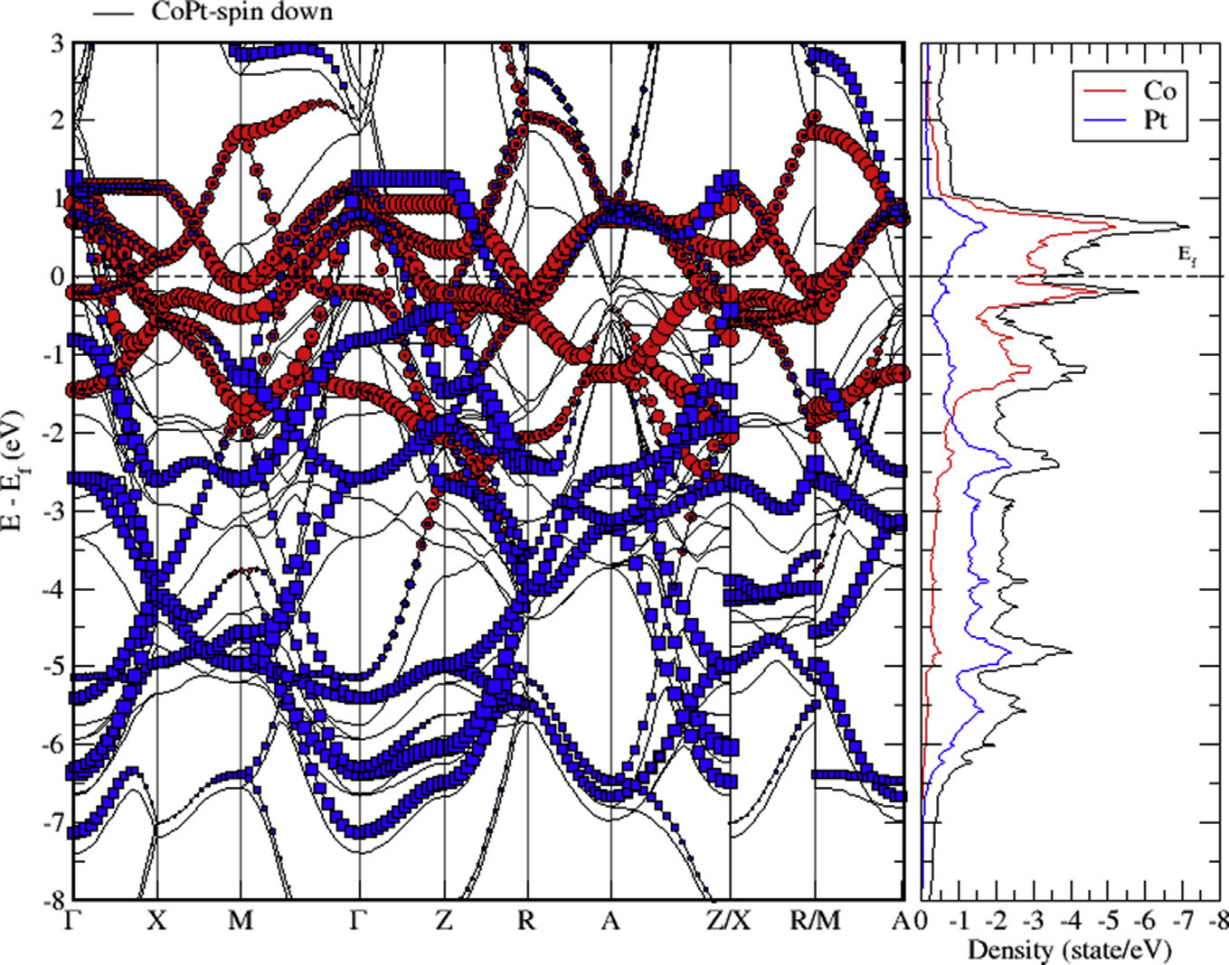
Electronic band structure and partial DOS of spin down states of CoPt binary.

The electronic band structure of CoPt is shown in Figs. 9, 10, and 11. As can be seen from Fig. 9, CoPt binary exhibits zero band gap. Figs. 10 and 11 indicate that within energy window (1 to -3 eV) around the Fermi level, DOS is dominated by the hybridization of Co-3d and Pt-5d states. The contribution to the conduction band is dominated by spin down electronic states. Moreover, within the energy window (-3 to -8 eV) below the Fermi level, the Pt-5d electronic states contribute significantly to the valence band. In addition, it is obvious that the degree of hybridization between Co-3d and Pt-5d electronic states is stronger for spin up than for spin down states.

#### 3.2.2 Density of states and electronic band structure of NiPt system

**Fig. 12:**
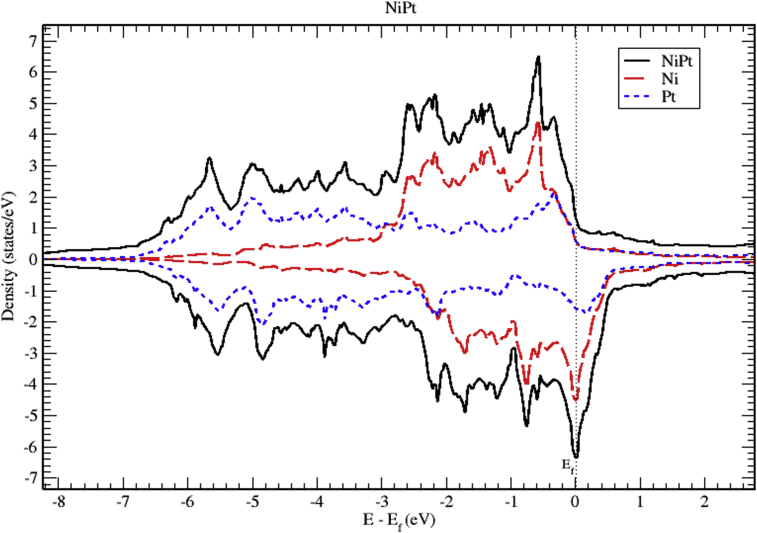
Total and partial density of states of NiPt binary, Ni (red) and Pt (blue).

**Fig. 13:**
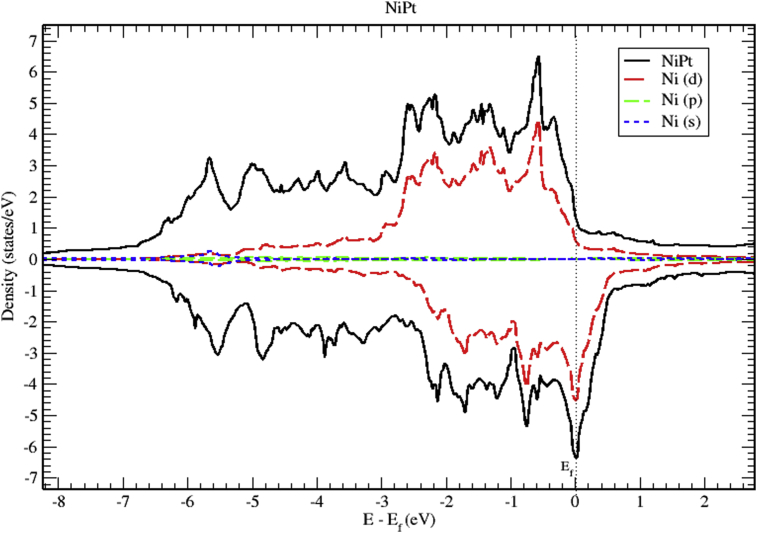
Total and partial density of states of NiPt binary, Ni-d (red), Ni-p (green) and Ni-s (blue).

**Fig. 14:**
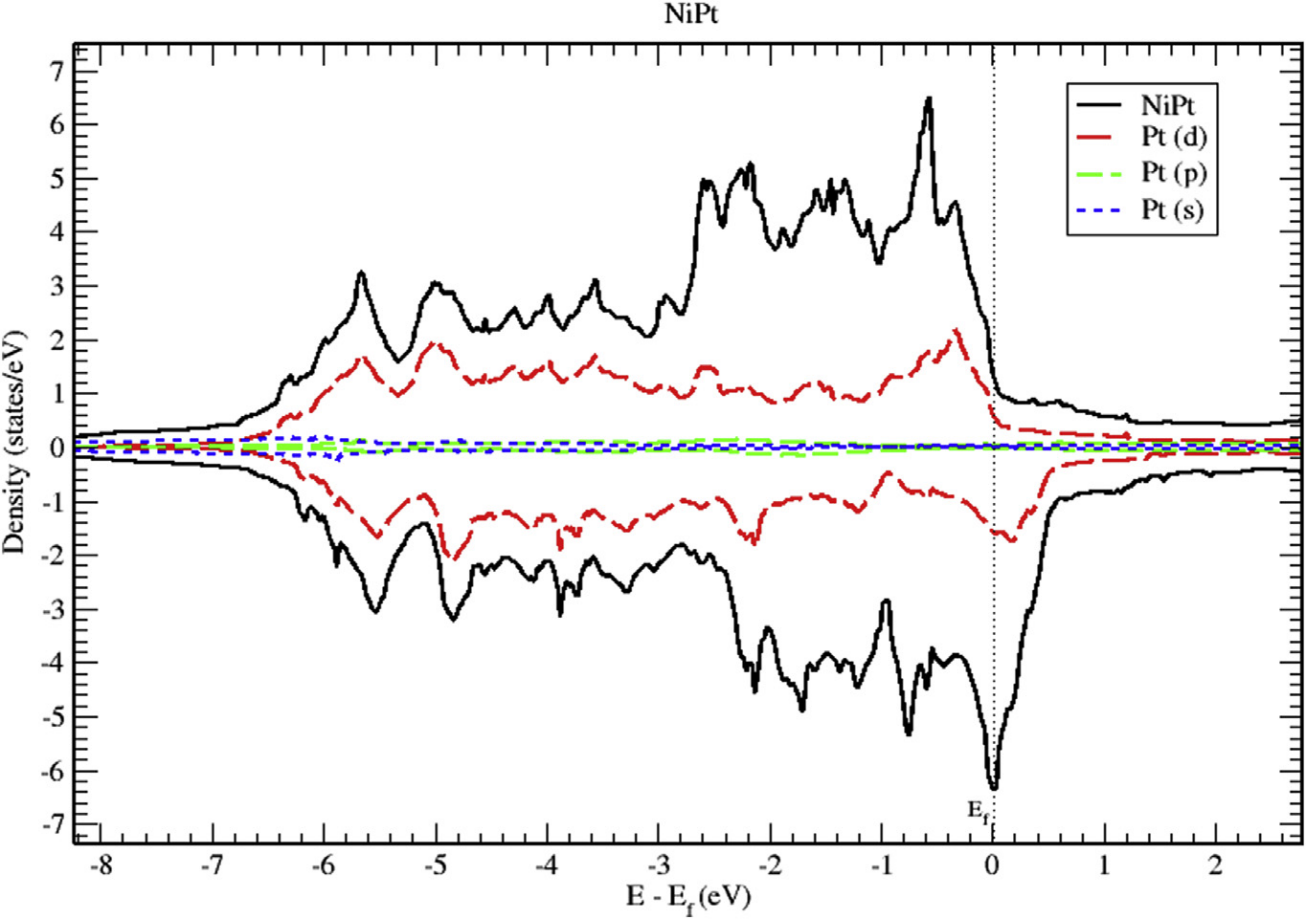
Total and partial density of states of NiPt binary, Pt-d (red), Pt-p (green) and Pt-s (blue).

The total and partial DOS of NiPt binary are displayed in Figs. 12, 13, and 14. Mainly, The d bands, with almost completely occupied bands of electrons dominate the valence band. On the other hand, the region slightly above Fermi level is partially occupied with spin down electrons. The total DOS of NiPt binary is characterized mainly by three spin up peaks refer to the highest DOS of spin up valence bands located at (-0.59, -2.15 and -2.58) eV below Fermi level, and four spin down peaks refer to the highest DOS of spin down bands located at (-0.03, -0.76, -1.70 and -2.13) eV below fermi level. One can see that the spin up band (5.95 eV) is narrower than spin down band (6.5 eV).

**Fig. 15:**
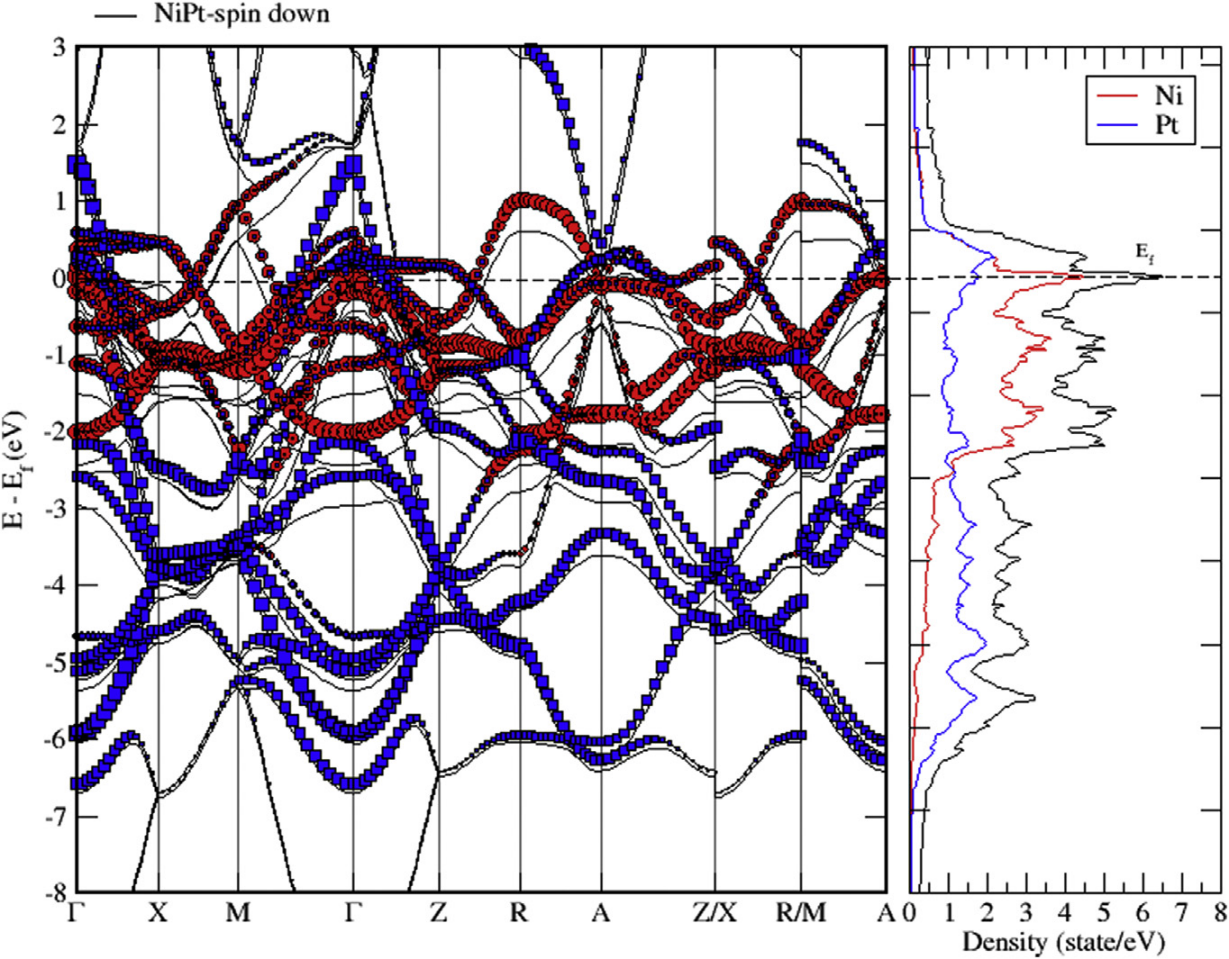
Electronic band structure and partial of NiPt binary.

**Fig. 16:**
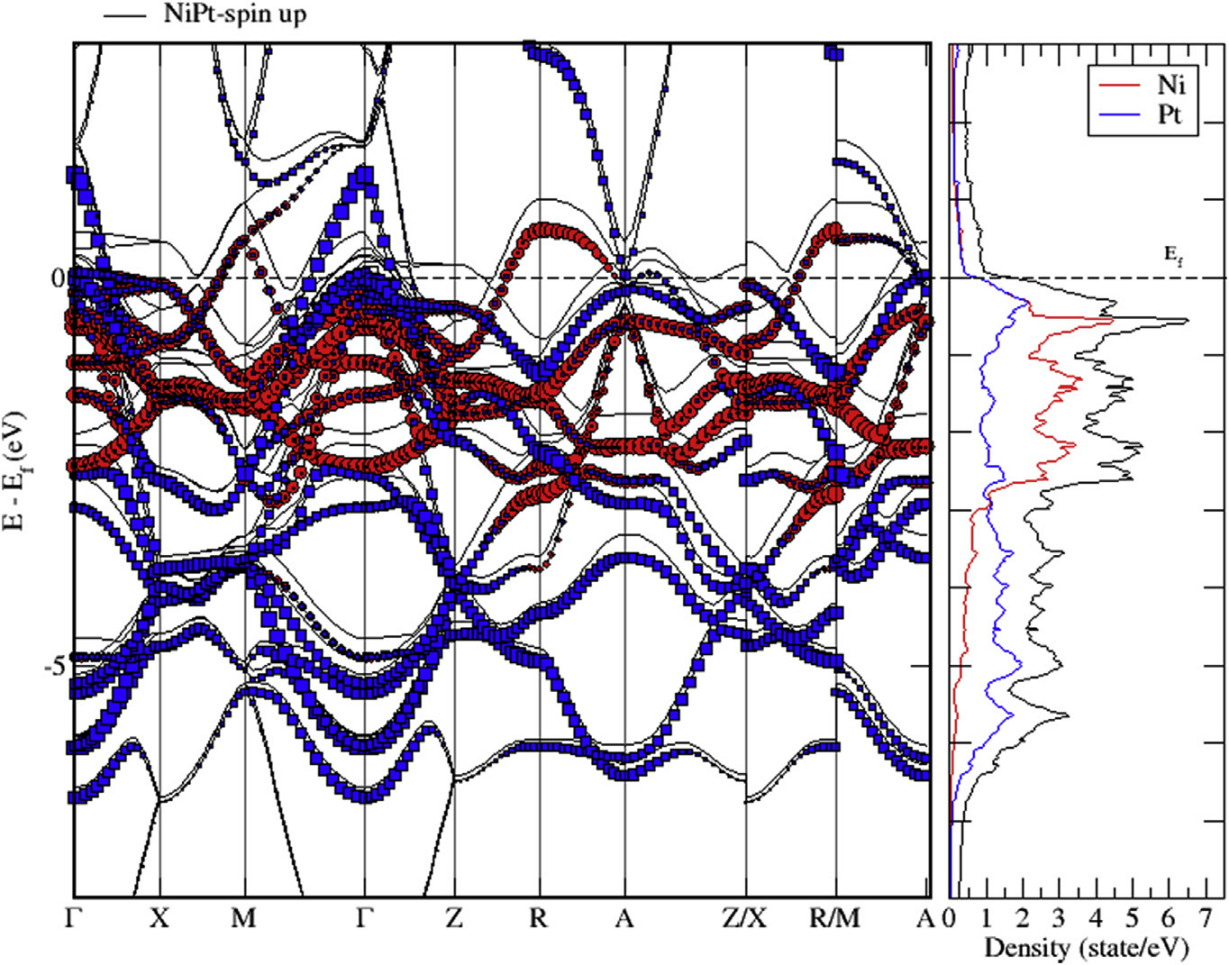
Electronic band structure and partial DOS of spin up states of NiPt binary.

**Fig. 17:**
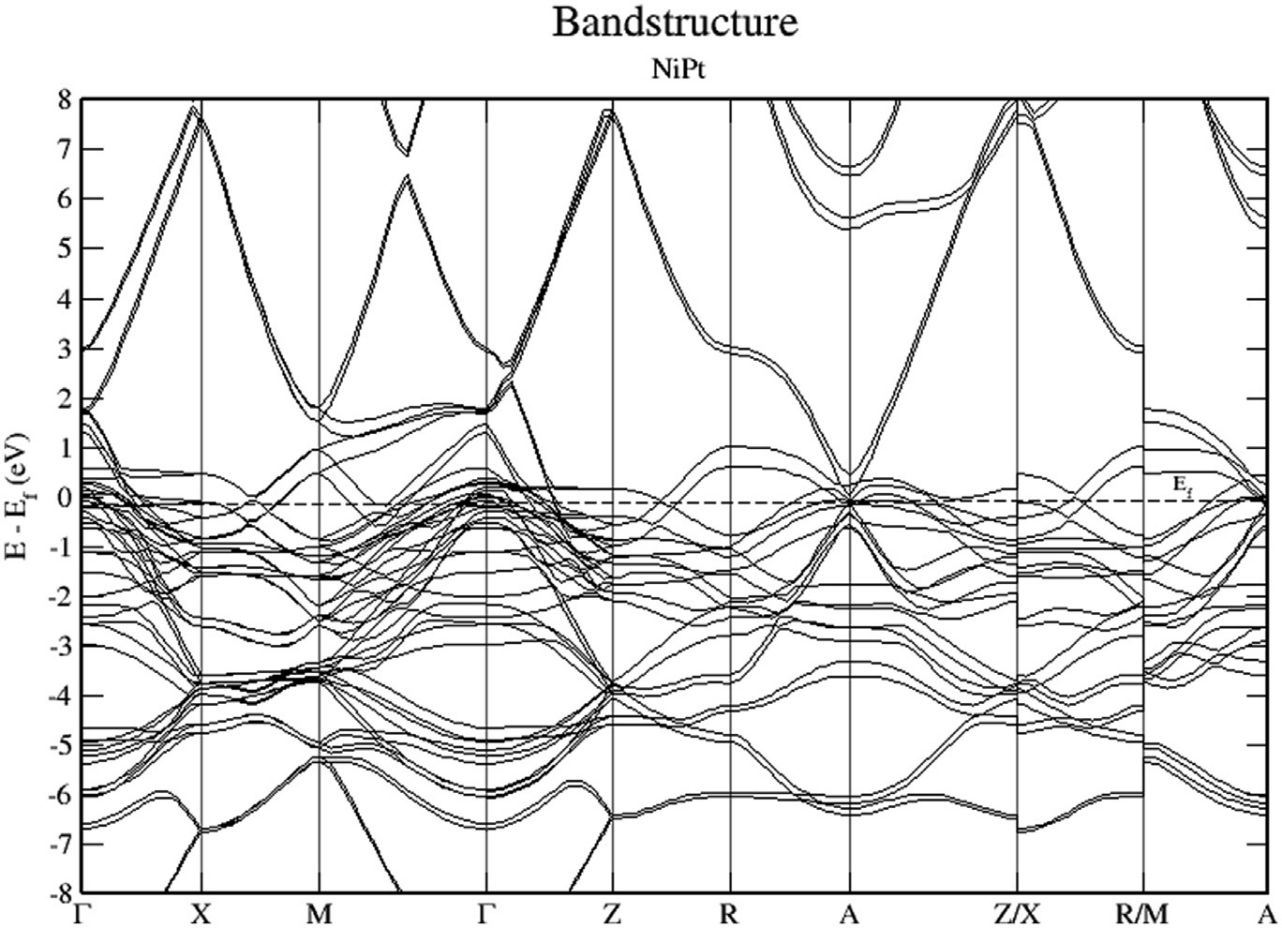
Electronic band structure and partial DOS of spin down states of NiPt binary.

Figs. 15, 16, and 17 show the band structure of NiPt binary. According to Fig. 15, the NiPt binary exhibits metallic nature. The density of states and band structure results indicate that within energy window (-2.5 - 0.5 eV) around the Fermi level both are dominated by the hybridization of Ni-3d and Pt-5d states and the conduction band is dominated by spin down states. Furthermore, within the energy window (-3 to -7 eV) below the Fermi level, the Pt-5d electronic states contribute significantly to the valence band. In addition, it is obvious that the spin down states are slightly shifted toward the higher energy levels.

#### 3.2.3 Density of states and electronic band structure of VPt_3_ binary

**Fig. 18:**
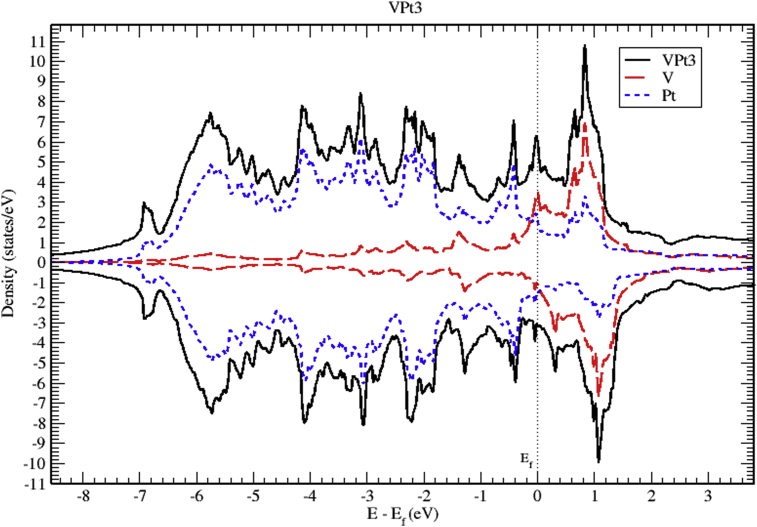
Total and partial density of states of VPt_3_ binary, V (red) and Pt (blue).

**Fig. 19:**
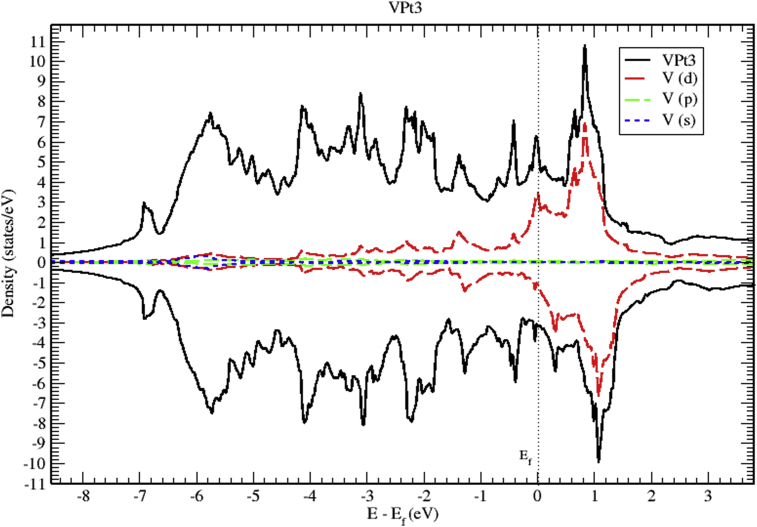
Total and partial density of states of VPt_3_ binary, V-d (red), V-p (green) and V-s (blue).

**Fig. 20:**
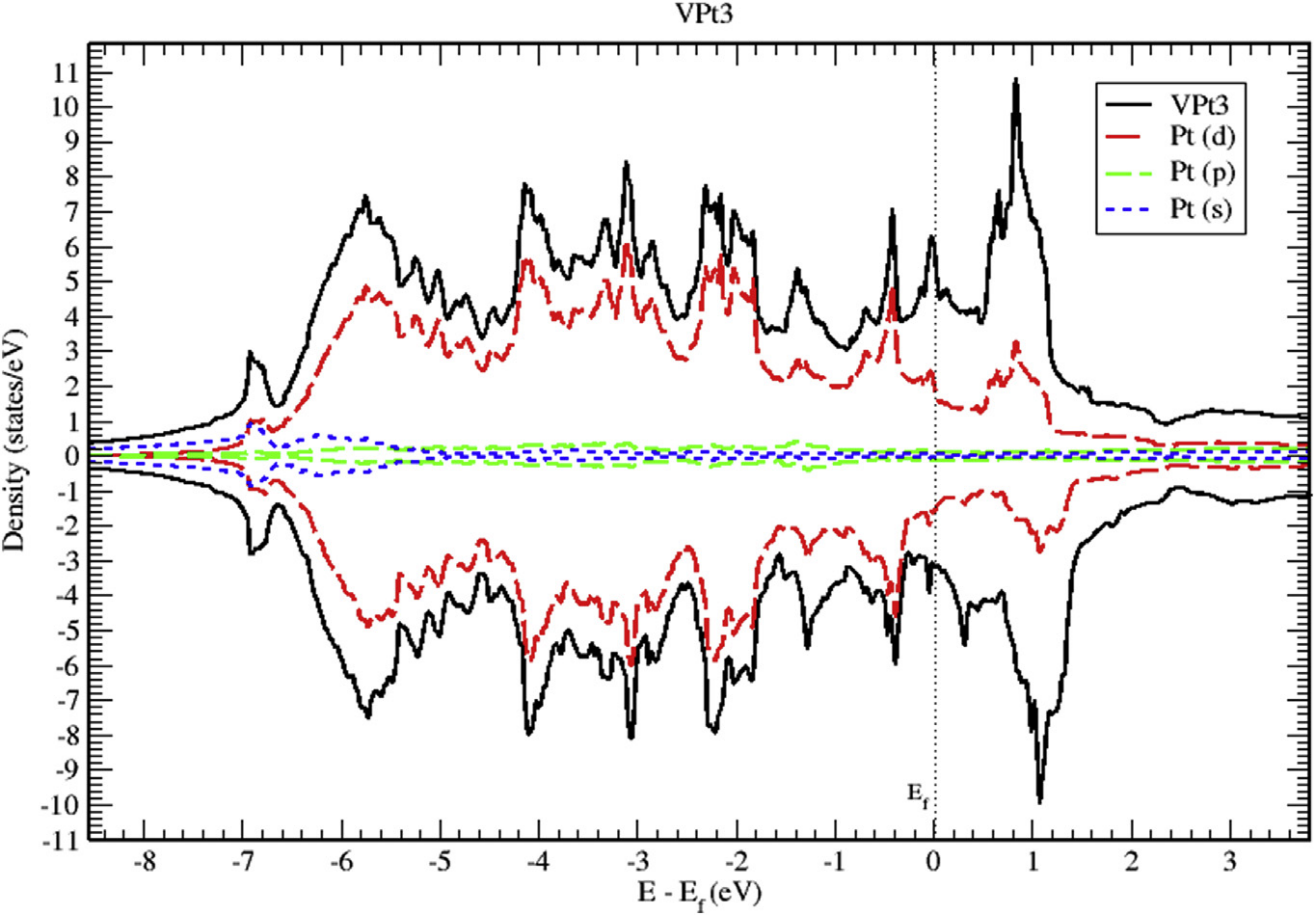
Total and partial density of states of VPt_3_ binary, Pt-d (red), Pt-p (green) and Pt-s (blue).

The total and partial DOS of VPt_3_ system are shown in Figs. 18, 19, and 20. The conduction band is constituted of the mixture of V-3d and Pt-5d electronic states with a major contribution from V-3d states. Furthermore, the valence band is almost fully occupied and composed mainly of Pt-5d electronic states. As can be seen from the figures, the profile of total DOS is similar for spin up and spin down states. The highest DOS of spin up bands is found to be located at 0.8 eV above Fermi level whereas the highest DOS of spin down bands is located at 1 eV above Fermi level.

**Fig. 21:**
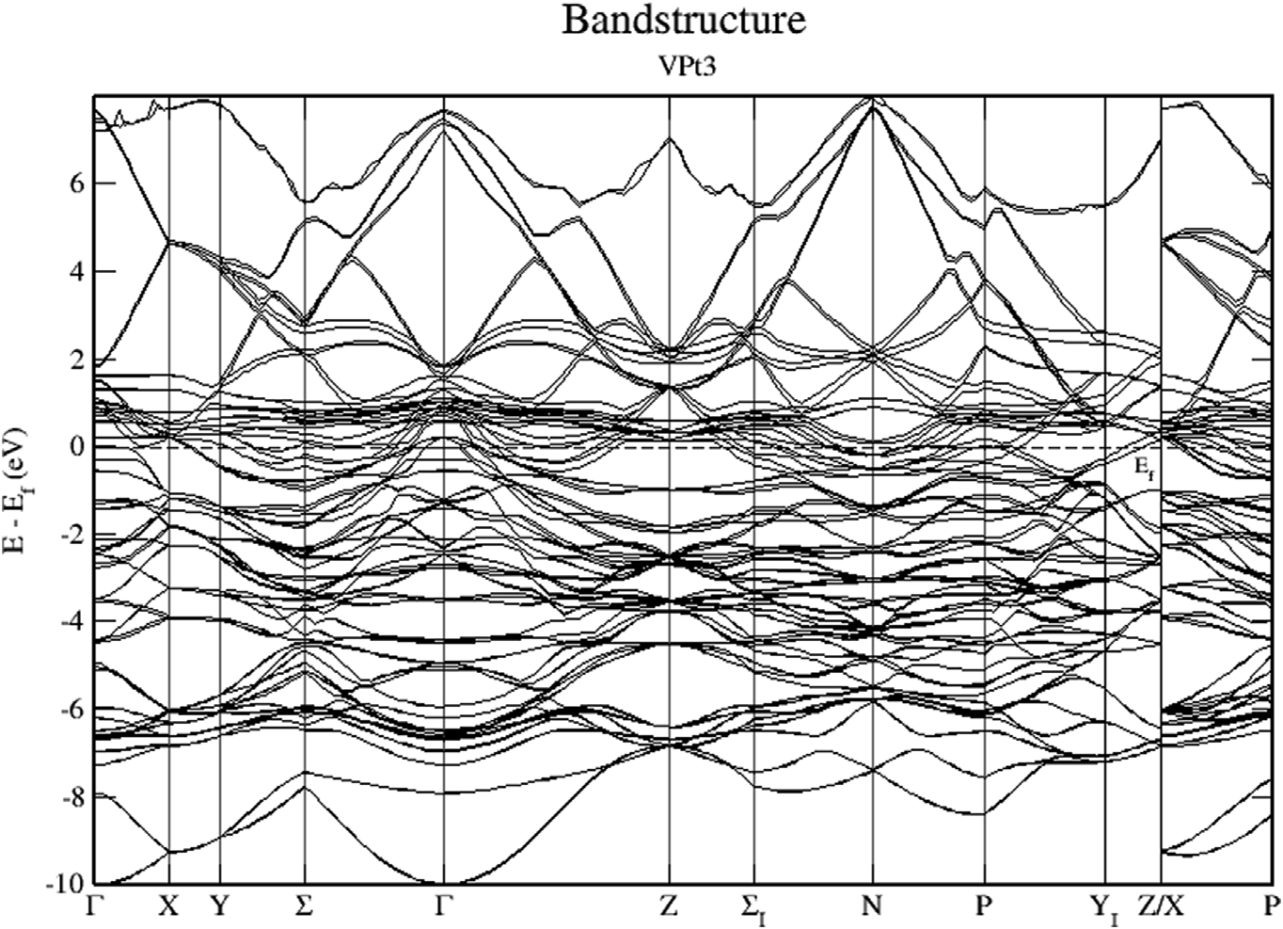
Electronic band structure of VPt_3_ binary along the symmetry path.

**Fig. 22:**
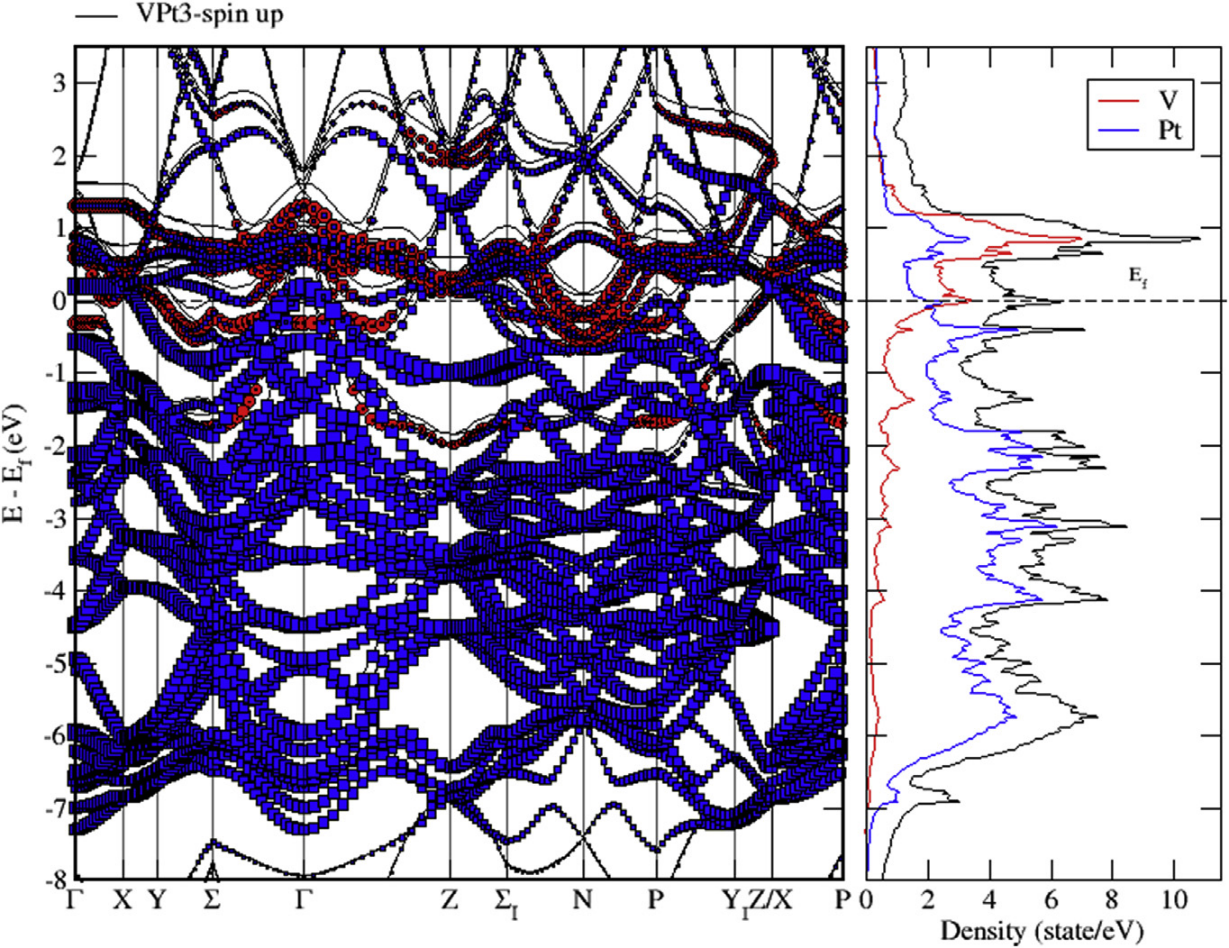
Electronic band structure and partial DOS of spin up states of VPt_3_ binary.

**Fig. 23:**
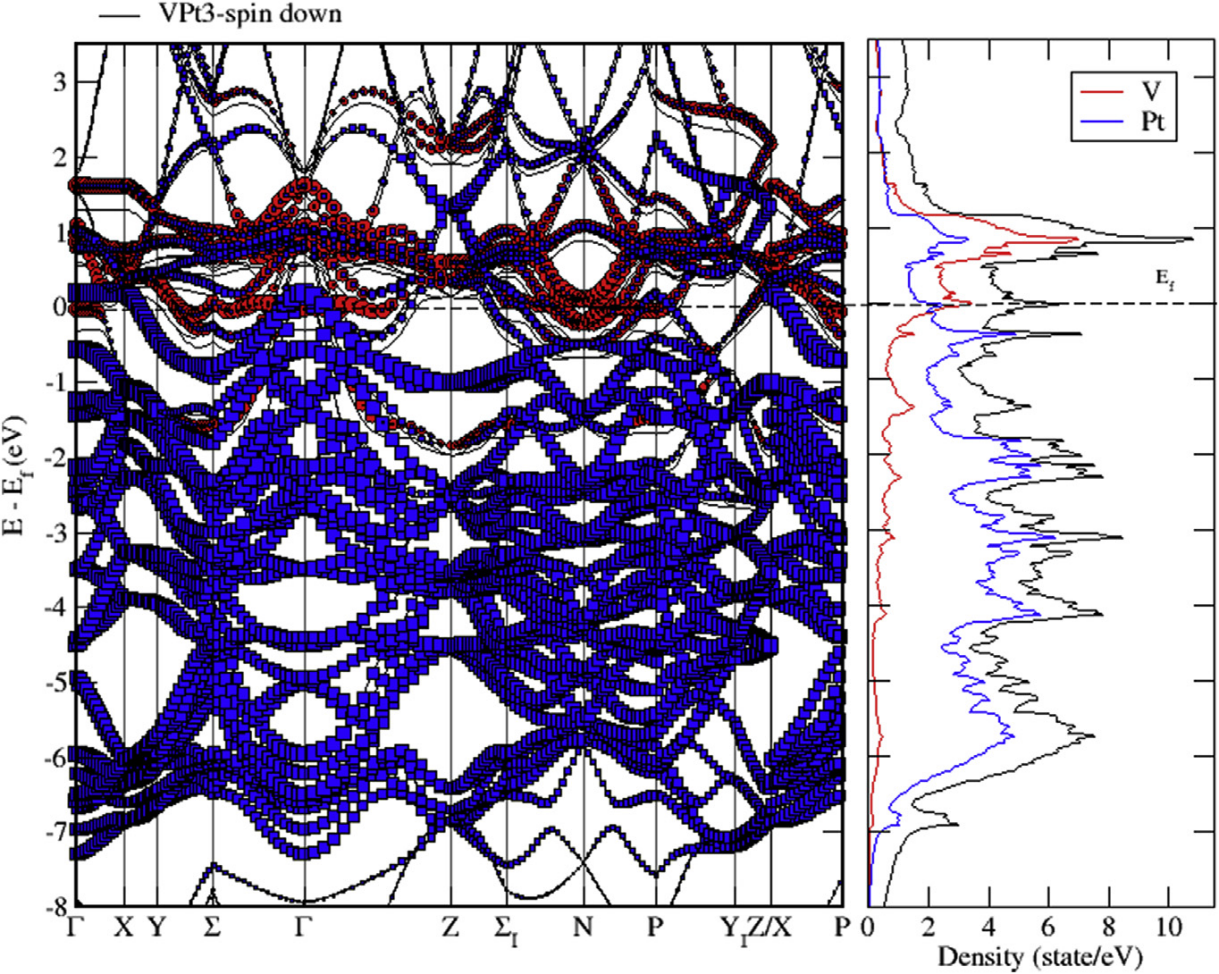
Electronic band structure and partial DOS of spin down states of VPt_3_ binary.

Figs. 21, 22, and 23 show the schematics of the band structure of VPt_3_. Careful examination of band structure and density of states plots indicate that within energy window (- 1 to 1 eV) around the Fermi level, DOS and band structure are dominated by the hybridization of Ni-3d and Pt-5d states. Moreover, within the energy window (-1 to -7 eV) below the Fermi level, the Pt-5d electronic states contribute significantly to the valence band. Clearly, the spin down states are slightly shifted toward the higher energy levels.

### 3.3 Determination of magneto-crystalline anisotropy energy (MAE)

Magneto-crystalline anisotropy energy (MAE) is an important parameter that characterizes the magnetic properties of a ferromagnetic material; it is an intrinsic property of a ferromagnetic material, which originates mainly from spin-orbit interaction. MAE can be defined as “the required energy to switch crystal magnetization from the favorable direction to other spatial directions”. From fundamental and technological points of view and depending on specific application, ferromagnetic materials with different values of MAE would be required. For instance, in applications such as information storage media, magnetic recording heads and permanent magnets, ferromagnetic materials with high MAE would be very useful.

Accurate calculation of MAE involves mainly the introduction of the spin-orbit interaction. Most calculations use either the ‘‘force theorem’’ [38] or a variational approach [39]. All materials investigated in this work exhibit tetragonal structures with uniaxial anisotropy [40, 41]. For such materials, the most appropriate method for the calculation of MAE is force theorem. This theorem states that the spin-orbit coupling prompted MAE energy is given by the difference in the relativistic band energies between two magnetization directions. The force theorem and LSDA are jointly implemented to calculate the MAE of all the binary structures investigated in this work. The validity of using this approach is justified by the fact that the charge- and spin-density variations caused by spin-orbit coupling disappear to first order in the spin-orbit coupling strength indicating that the spin-orbit term is accurately calculated [42].

The MAE is taken to be the difference in band energies of the two magnetization directions with spin-orbit coupling included in the Kohn-Sham equation while using the same self-consistent scalar-relativistic potential [43]. Two sets of self-consistent calculations will be performed for total energy where magnetic moments oriented parallel “*E*_∥_” and perpendicular” *E*_⊥_ “to the easy axis (c-axis) to determine the MAE in terms of the difference in these total energies [44]:

*MAE* = *E*_∥_ – *E*_⊥_.

MAE is one of the most important physical properties of the magnetic materials. Accurate calculation of this parameter is critical since this parameter determines how would the compounds be integrated into magneto-optical devices operating in a given energy window for some very sophisticated magnetic applications such as magneto-optical recording heads and information storage media in transformers. As described in the methodology section, the force theorem has been applied to calculate the magneto-crystalline anisotropy energy MAE for each of the three novel binary compounds. The MAE is taken to be the difference in band energies of the two-magnetization directions. The calculated MAE, magneto-crystalline anisotropy constant K and total magnetic moment values, are shown in Table 2.

**Table 2:** The PDOS occupancy of CoPt, NiPt and VPt_3_ binaries.

Magnetic Binary	Occupancy	PDOS	Total magnetic moment (μB)
CoPt	Co: 3d^7^ 4s^2^ Pt: 5d^9^ 6s^1^	Co: 3d Pt: 5d, 6s	4.31
NiPt	Ni: 3d^8^ 4s^2^ Pt: 5d^9^ 6s^1^	Ni: 3d Pt: 5d, 6s	1.86
VPt_3_	V: 3d^3^ 4s^2^ Pt: 5d^9^ 6s^1^	V: 3d Pt: 5d, 6s	0.8

Our results indicate that the DOS of CoPt and NiPt binaries of states have rather similar shapes for both spin directions and are only shifted with respect to each other by the exchange splitting. Fcc Ni has a similar density of states as Co, but with a smaller exchange splitting. The majority states for Co and Ni are fully occupied, whereas in the minority states 1.7 electrons are missing for Co and 0.6 electrons for Ni. This leads to magnetic moments of 1.8 μB per Co atom and 0.8 μB per Ni atom. Table 2 illustrates the occupancy of the PDOS of each of the investigated magnetic binaries. As can be seen from the table, the magnetic moment of CoPt is four times larger than the magnetic moment of NiPt. This can be attributed to the fact that Co-3d state has one unpaired electron per atom and thus Co-3d can have strong d-d hybridization with Pt-5d, while Ni-3d state has a weaker d-d hybridization with Pt-5d state because it has no unpaired electron. The third binary VPt_3_ has a vanishingly small magnetic moment at it exhibits tetragonal (TiAl_3_- like) crystalline structure which is known to posses weak magnetic moment.

Table 3 shows that the calculated moments are remarkable, in view of the fact that the only input to these calculations are the atomic number and the crystal structures. The majority states for CoPt and NiPt binaries are completely occupied, there are some empty majority states in VPt_3_ binary. This difference has been traditionally regarded as strong magnetism for CoPt and NiPt and weak magnetism for VPt_3_. This is consistent with the values of magnetic moments, MAE and K values as can be obviously seen from Table 2. One can clearly see that the K value of CoPt binary is an order of magnitude larger that the corresponding value of NiPt binary. In addition, the K value of CoPt binary is two orders of magnitude larger than the corresponding value of VPt_3_ binary. The calculated MAE and K match well with the available experimental observations or other theoretical predictions.

**Table 3:** The calculated value of MAE, anisotropy constant K and total magnetic moment of CoPt, NiPt and VPt_3_ binaries.

System	CoPt	NiPt	VPt_3_
MAE (meV/unit cell)	1.60	0.231	0.0116
MAE_th_ (meV/unit cell)	1.64 [45]	0.239 [46]	–
MAE_exp_ (meV/unit cell)	1.583–1.665 [47, 48, 49]	–	–
Anisotropy constant K (erg/cm^3^)	4.8 ×10^7^	6.9 ×10^6^	1.46 ×10^5^
Total magnetic moment (μ_B_)	4.31	1.86	0.8

## 4 Conclusions

In summary, ab intio calculations based on spin density functional theory within the framework of generalized gradient approximation GGA and local spin density approximation LSDA have been carried out to investigate the structural, optical, electronic and magnetic properties of M_x_Pt_1-x_ (M = Co, Ni and V) magnetic systems. The equilibrium, in-plane lattice constant (*a*_*o*_)^,^ out-off plane lattice constant (*c*_*o*_) and the axial ratio (*c*_*o*_*/a*_*o*_) were calculated. We found that *a*_*o*_, *c*_*o*_ and *V*_*o*_ for CoPt, NiPt and VPt_3_ binaries are equal to (*a*_*o*_ = 3.806 A, *c*_*o*_ = 3.707 A, *c*_*o*_/*a*_*o*_ = 0.97 and *V*_*o*_ = 53.7 A^3^) (*a*_*o*_ = 3.84 A, *c*_*o*_ = 3.62 A, *c*_*o*_*/a*_*o*_ = 0.94 and *V*_*o*_ = 53.64 A^3^) and (*a*_*o*_ = 3.88 A, *c*_*o*_ = 7.88 A, *c*_*o*_*/a*_*o*_ = 2.03 and *V*_*o*_ = 118.71 A^3^) respectively. In addition, the electronic band structure, density of states and partial density of states have been investigated to obtain a deeper understanding of the optical and electronic properties of the three investigated magnetic binaries.

Using total energy minimizations performed for each binary. We found that the most stable phase of CoPt and NiPt binaries is the distorted face centered tetragonal L1_0_ structure whereas the most stable phase of VPt_3_ is the D0_22_ tetragonal TiAl_3_- like crystalline structure.

Analysis of band structure data indicates that CoPt, NiPt and VPt_3_ magnetic binary systems are metallic in nature as indicated by the obtained zero band gap for each. To elucidate the contribution of each ionic species in optical and electronic properties, we calculated the total density of states and partial density of states of each binary. We found that the major contribution to the total density of state comes from hybridization of M-3d and Pt-5d states. This result is expected when transition metals are mixed with Pt. The predominance of this kind of hybridization is expected to enhance both spin and orbital magnetic moments and thus would improve the magnetic properties of the three binary magnetic systems investigated in this work. We found that this strong hybridization in the energy range for CoPt (-0.2 to -3.5) eV below Fermi level, has enhanced the magnetic moments and thus the magneto-crystalline anisotropy energies of CoPt to a large extent. Furthermore, the smaller hybridization between Ni-3d and Pt-5d states of NiPt in the energy range (0.2 to -2.6) eV below Fermi level leads to a smaller value of magnetic moment and MAE values. A further reduction of the hybridization between V-3d and Pt-5d states in the (0–1) eV energy range above Fermi level leads to a further decrease in the magnetic moment and MAE values of the VPt_3_ binary.

We found that MAE value of CoPt is 1.60 meV/unit cell corresponds to magneto-crystalline anisotropy constant K equals to 4.8 × 10^7^ erg/cm^3^. In addition, we found the total magnetic moment of this phase is 4.31 μB^.^ Furthermore, we found that MAE value of NiPt is 0.231 meV/unit cell corresponds to magneto-crystalline anisotropy constant K equals to 6.9 × 106 erg/cm^3^ and the total magnetic moment of this phase is 1.86 μB. However, we found an extremely small MAE 0.0116 meV/unit cell and K 1.46 × 105 erg/cm^3^ values for the VPt_3_ indicated by the obtained vanishingly small magnetic moment 0.80 μB of this binary. The smaller value of MAE in NiPt and VPt_3_ compared to CoPt is consistent with the exchange splitting found in the three magnetic binaries.

Based on our findings, L1_0_ phase of CoPt may be a potential candidate for the fabrication of magneto-optical devices, magnetic actuators, ultrahigh density magnetic storage and drivers in micro- and nano-electromechanical systems. To a smaller extent, L1_0_ NiPt could be used for certain magnetic applications such as magnetic storage and perpendicular magnetic data recording. The small values of MAE and K of VPt_3_ D0_22_ tetragonal phase make it eligible for building blocks of motors, generators, inductors, transformers and sensors as a soft magnetic material.

## Declarations

### Author contribution statement

A. M. Alsaad: Conceived and designed the experiments; Performed the experiments; Analyzed and interpreted the data; Contributed reagents, materials, analysis tools or data; Wrote the paper.

A. A. Ahmad: Conceived and designed the experiments; Analyzed and interpreted the data; Contributed reagents, materials, analysis tools or data; Wrote the paper.

H. A. Qattous: Conceived and designed the experiments; Performed the experiments; Contributed reagents, materials, analysis tools or data.

### Funding statement

This work was supported by the deanship of scientific research at Jordan university of science and technology.

### Competing interest statement

The authors declare no conflict of interest.

### Additional information

No additional information is available for this paper.
